# Pulmonary hypertension in a hemodialysis porcine model: possible unforeseen causes?

**DOI:** 10.1080/0886022X.2022.2064305

**Published:** 2022-06-07

**Authors:** Ákos Pethő, János Szebeni

**Affiliations:** a Department of Internal Medicine and Oncology, Semmelweis University, Budapest, Hungary; b SeroScience Ltd, Budapest, Hungary; c Department of Translational Medicine, Nanomedicine Research and Education Center, Semmelweis University, Budapest, Hungary

Dear Editor,

We read with great interest the letter to the editor from Karim M Soliman, Vinaya Rao, and Ahmed Daoud’s entitled ‘Pulmonary Hypertension in a Hemodialysis Porcine Model: Possible Unforeseen Causes’. Based on the known vasoconstrictive effect of low pH, the authors raise the possibility that the low (5.5) pH of the 200-250 mL physiological saline ‘rinsed back’ into the body at the end of the dialysis could be an unforeseen cause of pulmonary hypertension that we described. In fact, rather than establishing the cause of this observation, we came up with multiple potential explanations, so we appreciate the authors' attempt to think together with us on this puzzling finding. However, their proposal also raises questions because the way the study was conducted is not consistent with the above ‘acidification theory’. The dialysis tubing and the circulation were a ‘closed system’, implying that the only time when the whole amount of the saline, pre-filled into the extracorporeal tubing, reached the circulation was at the beginning of the experiment. Then, the animal's blood ‘washed’ the saline from the tubing into the circulation, at which time we did not detect pulmonary hypertension. On the other hand, when we detected pulmonary hypertension, the animal's own blood was pushed back into the body from the tubing, using saline; the two had no change to equilibrate, the blood could not be diluted to a degree that would bring down its pH to that of saline. Even if a small amount of saline got into the circulation at the end of the rinse-back procedure, it is very unlikely that it could cause a significant sudden drop in the blood's pH. So, we believe, the cause of pulmonary hypertension remains open for further ideas, which we appreciate very much.

